# Serum Immunoglobulin Free Light Chain Assessment in IgG4-Related Disease

**DOI:** 10.1155/2013/426759

**Published:** 2013-06-26

**Authors:** Aurélie Grados, Mikael Ebbo, José Boucraut, Frédéric Vély, Pierre Aucouturier, Aude Rigolet, Benjamin Terrier, David Saadoun, Pascale Ghillani-Dalbin, Nathalie Costedoat-Chalumeau, Jean Robert Harlé, Nicolas Schleinitz

**Affiliations:** ^1^Service de Médecine Interne, Centre Hospitalier Universitaire Hôpital de la Conception, AP-HM, Aix-Marseille Université, 13385 Marseille Cedex 5, France; ^2^Laboratoire d'Immunologie, Centre Hospitalier Universitaire Hôpital de la Conception, AP-HM, Aix-Marseille Université, 13385 Marseille Cedex 5, France; ^3^INSERM, UMR-S 938, Hôpital Saint-Antoine, Université Pierre et Marie Curie, 75012 Paris, France; ^4^Service de Médecine Interne, Hôpital Pitié-Salpétrière, AP-HP, Université Pierre et Marie Curie, 75013 Paris, France; ^5^Laboratoire d'Immunologie, Hôpital Pitié-Salpétrière, AP-HP, Université Pierre et Marie Curie, 75013 Paris, France

## Abstract

Immunoglobulin free light chains are produced in excess during normal antibody synthesis. Their evaluation is commonly used in case of a monoclonal gammopathy. In polyclonal hypergammaglobulinemia related to the Sjögren syndrome or systemic lupus, erythematosus serum free light chain levels are increased and could correlate with disease activity. We show here that the **κ** (*P* < 0.0001) and **λ** (*P* = 0.0003) free light chains and the **κ** : **λ** ratio (*P* = 0.0049) are increased in sixteen patients with IgG4-related disease when compared to healthy controls. The increase of **κ** and **λ** free light chains probably reflects the marked polyclonal B cell activation of the disease. We could not assess in this small cohort of patients a significative correlation of serum free light chain levels and disease activity or extension.

## 1. Introduction

Immunoglobulin free light chains (FLCs) are produced during antibody synthesis by B lymphocytes. Immunoglobulins have a tetrameric structure composed of two identical heavy and two identical light chains (kappa or lambda) linked together by disulphide bonds. Heavy and light chains assembly occurs in the endoplasmic reticulum. During antibody synthesis, there is an excess of light chain production. These FLCs are secreted into the circulation, where rapid renal clearance results in a short half-life of 2–6 hours [1]. Nowadays highly sensitive nephelometric immunoassay, with antibodies recognizing epitopes specific for free kappa (*κ*) or lambda (*λ*) light chains, are available [2]. Reference and diagnostic ranges for serum FLC (sFLC) and the *κ* : *λ* ratio have been determined with these assays [3]. In the context of a monoclonal gammopathy of undetermined significance the evaluation of the *κ* : *λ* ratio has been shown to correlate with the prognosis [4]. Moreover, this assay is now commonly used to quantify the monoclonal component in case of light chain multiple myeloma or AL amyloidosis [5, 6]. 

Decreased glomerular filtration rate (GFR) in renal insufficiency, associated either with a monoclonal gammopathy or other chronic kidney diseases, has an influence on FLC levels. Polyclonal sFLC levels are increased as a consequence of the reduction of their clearance [1]. The *κ* : *λ* ratio also increases because the *κ* FLC clearance is more influenced by the GFR than the *λ* FLC [1]. 

sFLC overproduction has also been reported in cases of a polyclonal increase of immunoglobulins. This has been shown in systemic lupus erythematosus (SLE), rheumatoid arthritis (RA) and Sjögren's syndrome [7–9]. However, in a context of polyclonal hypergammaglobulinemia, the *κ* : *λ* ratio remains unchanged [8]. In some of these autoimmune diseases, *κ* or *λ* sFLC levels have been shown to correlate with disease activity and to vary under treatment [7, 9–11].

IgG4-related disease (IgG4-RD) is a systemic disease associated in most cases to hypergammaglobulinemia [12–14]. Tissues affected by this condition present with polyclonal lymphoplasmacytic infiltrate, fibrosis with a storiform pattern, obliterative phlebitis, and scarce eosinophils [15]. The polyclonal infiltrating plasmacytes are in majority IgG4 positive. The excessive skewed production of IgG4 is observed in 80–90% of patients on serum analysis and is retained as a diagnostic criterion [13]. IgG4 serum levels are currently the unique available biological marker of the disease. The B lymphocyte activation associated to polyclonal hypergammaglobulinemia and IgG4 production prompted us to investigate serum free *κ* and *λ* light chains in this condition. 

## 2. Patients and Methods

### 2.1. Patients

We retrospectively collected available frozen serum samples from IgG4-RD patients at two internal medicine departments in France. Most of the samples collected were serum from IgG4-RD patients at diagnosis before any treatment or at relapse. Serum after treatment was available for comparison for very few patients. Clinical, biological, radiological, and pathological characteristics of IgG4-RD were also collected.

### 2.2. Laboratory Analysis

Analysis was performed on frozen samples at −20°C. The stability of FLC for many years at −20°C has been demonstrated [16], and previous studies in autoimmune disease were performed on frozen samples [7]. Samples analyzed in this study were stored at −20°C for no more than 4 years.

sFLC levels were measured in the same laboratory by using a latex-enhanced immunoassay (Freelite, The Binding Site, Birmingham, UK) using a nephelometric analyzer (SPAplus, The Binding Site, Birmingham, UK) for IgG4-RD sample and healthy controls. The immunoassay consisted of two separate measurements, one to detect free *κ* (normal range: 3.3–19.4 mg/L) and the other to detect free *λ* (normal range: 5.7–26.3 mg/L). The diagnostic ranges had been previously established by the manufacturer to include 100% of a reference population of 282 serum samples. A ratio of *κ* : *λ* <0.26 or >1.65 is abnormal, according to the manufacturer's recommendations [2]. Values for GFR rate were only reported if GFR was less than 75 mL/min/1.73 m^2^. IgG4 (measured by nephelometry, The Binding Site, Birmingham, UK) and total IgG were analyzed in the same sample for IgG4-RD patients. Healthy controls were also analyzed by the same way.

### 2.3. Statistical Analysis

Values obtained for *κ*, *λ*, *κ* : *λ* ratio in IgG4-RD patients were compared with values from healthy controls. Values from patients with active disease (serum sample collected at diagnosis or relapse) were compared with values obtained in few patients with inactive disease. Comparison of distributions was performed by using the Mann-Whitney test. Differences were considered significant when *P* < 0.05. Correlation was evaluated using Spearman's test. Graphic representation and statistical analysis were obtained using GraphPad Prism software (San Diego, CA, USA). 

## 3. Results

### 3.1. Patients

Frozen serum was available for 22 patients but the analysis of *κ* and *λ* FLC levels failed in 6 because of small volumes. Results were available for analysis in 16 patients with IgG4-RD. The characteristics of the patients and the state of the disease at the time of sample collection are given in Table 1. All patients had histological confirmation of IgG4-RD except patient no. 9 presenting with abdominal periaortitis and IgG4 elevation in serum. There was a majority of males (15 males for 1 female) and the mean age was 67 years (range 27–84 years). Only 4 patients had a unique organ involved by IgG4-RD. The majority of the patients had more than one organ involved (ranging from 2 to 8). Renal insufficiency defined by a glomerular filtration rate <75 mL/min/1.73 m^2^ was reported for 4 out of 16 IgG4-RD patients and one of these patients required hemodialysis at the time of sample collection. The *κ* and *λ* FLC levels were also analyzed in 14 healthy control patients.

### 3.2. sFLC Assessment in IgG4-RD Patients

*κ* and *λ* FLC levels could be assessed in 16 patients. Of these, 15 had active disease (sample at diagnosis or at relapse before treatment) and 1 was only analyzed after treatment during inactive disease. Samples from 3 patients were available before and after treatment for comparison. 

**Figure 1:**
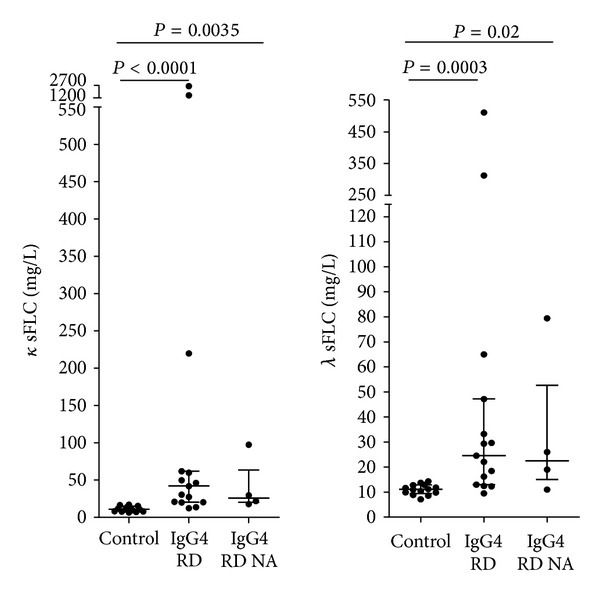
Kappa/lambda serum FLC in patients with IgG4-RD. Comparison of serum-free *κ* and *λ* light chains in controls, IgG4-RD patients with active disease (IgG4-RD) and nonactive disease (IgG4-RD NA). Median and interquartile ranges are shown.

All results are given as median with interquartile ranges. The *κ* and *λ* FLC levels were significantly higher (*P* < 0.0001 and *P* = 0.0003) in IgG4-RD patients than in healthy controls (Figure 1).

IgG4-RD patients at diagnosis before treatment or at relapse (*n* = 15) had median *κ* FLC of 41.97 mg/L (20.39–61.85) versus 10.75 mg/L (7.82–14.8) in healthy controls. Only 2 out of these 15 patients (13%) had normal *κ* FLC levels (normal range fixed by the manufacturer 3.3 < *κ* < 19.4 mg/L). All other patients had increased *κ* FLC levels. In IgG4-RD patients analyzed during inactive disease (*n* = 4) median 25.77 (20–63.42) *κ* FLC levels were lower, but also significantly higher than in controls (*P* = 0.0035). However, the difference of *κ* FLC levels between patients with active or inactive disease was not statistically significant (*P* = 0.65). When excluding patients with renal insufficiency (GFR < 75 mL/min/1) the comparison of *κ* FLC levels between controls and IgG4 RD patients (*n* = 12) with active disease and inactive disease (*n* = 3) remained significant (*P* = 0.0001 and *P* = 0.009). 

The comparison of the *κ* sFLC in three patients between active and nonactive disease showed a decrease in two patients (patient no. 1 and patient no. 5) and an increase in the third (patient no. 6).

Median *λ* FLC levels were of 24.54 mg/L (13–47.2) in the 15 IgG4-RD patients with active disease versus 11.1 mg/L (9.34–14.3) in healthy controls. Eight out of these 15 patients (53%) had normal *λ* FLC levels (normal range fixed by the manufacturer: 5.7 < *λ* < 26.3 mg/L). All other patients had increased *λ* FLC levels. The comparison of *λ* FLC levels between controls and IgG4-RD patients with inactive disease, median 22.51 mg/L (51.02–52.71), was also statistically significant, *P* = 0.02. The comparison between IgG4-RD patients with active or inactive disease was not significant for *λ* FLC levels, *P* = 0.8. When excluding patients with renal insufficiency (GFR < 75 mL/min/1.73 m^2^), the comparison of *λ* FLC levels between controls and IgG4 RD patients (*n* = 12) with active disease remained significant (*P* = 0.001). However, the significance was lost with patients with inactive disease (*n* = 3) (*P* = 0.08).

The comparison of the *λ* sFLC in three patients between active and nonactive disease showed a decrease in one patient (patient no. 1) and an increase in two (patient no. 5 and patient no. 6).

Renal insufficiency, defined here as a GFR < 75 mL/min, was present in 4 out of the 16 patients analyzed for *κ* and *λ* FLC levels. Median *κ* FLC levels in IgG4-RD patients with renal insufficiency were of 799.2 mg/L (69.64–2056) versus 30.48 mg/L (20.27–54.83) in patients without renal insufficiency (*P* = 0.02). Median *λ* FLC levels in IgG4-RD patients with renal insufficiency were of 195.7 mg/L (54.37–411.5) versus 19.53 mg/L (12.38–31.44) in patients without renal insufficiency (*P* = 0.01). We also compared the sum of *κ* + *λ* sFLC levels between IgG4-RD patients and controls. There was a significant increase of median 71.28 mg/L (36.33–126.9) *κ* + *λ* sFLC levels in IgG4-RD patients with active disease when compared with controls 21.2 mg/L (17.6–27.6) (*P* < 0.0001).

None of these patients had a monoclonal gammopathy. Total IgG levels in IgG4-RD patients were significantly higher than in controls, median 15.09 g/L (13.2–20.29) versus 10.45 g/L (8.56–12.95) (*P* = 0.0003). Median IgG4 levels were of 5.94 (0.64–3.54) with (extremes 0.26–36.70 g/L) in patients. There was a significant correlation (*P* < 0.001, Spearman *r* = 0.84) between *κ* and *λ* FLC levels in IgG4-RD patients. There was no statistically significant correlation between the IgG4 levels and either *κ* sFLC (*P* = 0.21, Spearman *r* = 0.21) or *λ* sFLC (*P* = 0.25, Spearman *r* = 0.3) levels. There was a correlation of total IgG levels with *κ* sFLC (*P* = 0.006, Spearman *r* = 0.67) but not with *λ* sFLC (*P* = 0.3, Spearman *r* = 0.27). 

No correlation was evidenced in our cohort between FLC levels and demographic or clinical features of patients. *κ* and *λ* FLC levels were higher in patients with more than 3 organ involvement than in those with 3 or less (*κ* 49.8 mg/L (29.0–799.2) versus 20.9 mg/L (20.1–61.85), *P* = 0.2; *l* 29.63 mg/L (18.42–195.7) versus 18.4 mg/L (13.0–33.24), *P* = 0.2) but the difference was not statistically significant.

### 3.3. *κ* : *λ* Ratio Is Increased in a Significant Proportion of IgG4-RD Patients

Normal *κ* : *λ* ratio according to the manufacturer is comprised between 0.26 and 1.65. In patients with renal insufficiency the ratio can be higher and comprised between 0.37 and 3.01 [1].

**Figure 2:**
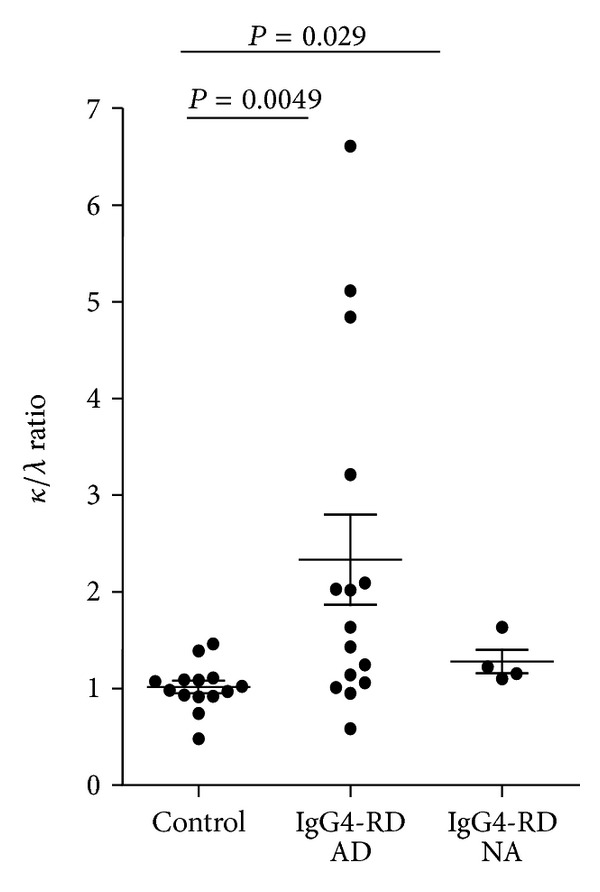
Kappa/lambda serum FLC ratio in patients with IgG4-RD. Comparison of serum free *κ* and *λ* light chains ratio in controls, IgG4-RD patients with active disease (IgG4-RD AD) and nonactive disease (IgG4-RD NA). Median and interquartile ranges are shown.

IgG4-RD patients with active disease had a significantly higher *κ* : *λ* FLC ratio than controls (Figure 2) with a median 1.63 (1.06–3.2) versus 1.00 (0.91–1.1) (*P* = 0.0049). The *κ* : *λ* FLC ratio was higher than the upper range of 3.01 in 2 out of 4 IgG4-RD patients with renal insufficiency (range 1.25–5.11). It was higher than the upper range of 1.65 in 5/12 (41.6%) IgG4-RD patients without renal insufficiency (range 2.09–6.6). Thus 7 out of 16 (43.7%) IgG4-RD patient presented with an increased abnormal *κ* : *λ* FLC ratio at diagnosis. The *κ* : *λ* FLC ratio was also statistically different when comparing IgG4-RD patients with inactive disease and controls (*P* = 0.029) but not with patients with active disease, *P* = 0.39. In both controls and IgG4-RD patients with inactive disease, all *κ* : *λ* FLC ratios were in the normal range.

When excluding patients with renal insufficiency (GFR < 75 mL/min/1.73 m^2^), the comparison of the *κ* : *λ* ratio between control and IgG4 RD patients (*n* = 12) with active disease remained significant (*P* = 0.02). However the significance was lost with patients with inactive disease (*n* = 3) (*P* = 0.05).

The comparison of the *κ* : *λ* ratio in three patients between active and nonactive disease showed a decrease in two patients (patient no. 1 from 2.02 to 1.63 and patient no. 5 from 3.21 to 1.1) with normalization of the ratio and a stability in the third (patient no. 6 from 1.01 to 1.15).

## 4. Discussion

An increased free light chain concentration has been reported in a variety of inflammatory and autoimmune diseases and reflects the polyclonal B lymphocytes activation in these pathologies. For example, an increase of the *κ* and *λ* sFLC levels or total (*κ* + *λ*) sFLC levels has been reported in systemic lupus erythematosus (SLE) [7], rheumatoid arthritis, and Sjögren's syndrome [8, 11]. Our results, obtained on a small cohort of IgG4-RD patients, show a significant increase of *κ* and *λ* sFLC levels as in these autoimmune diseases. 

The presence of a monoclonal gammopathy, that can be associated with an abnormal *κ* : *λ* ratio and an excess of either *κ* or *λ* FLC, was excluded here.

Serum FLC levels and the *κ* : *λ* ratio are also influenced by the glomerular filtration rate [1]. In this study, patients with renal insufficiency had significantly higher values of sFLC than other IgG4 RD patients. However, we show that the significant increase of the *κ* FLC, the *λ* FLC, and the *κ* : *λ* ratio is also observed in IgG4 RD patient with active disease and normal renal function (GFR > 75 mL/min/1.73 m^2^). Thus, renal insufficiency is not the only cause of FLC increase in IgG4 RD.

This increase of sFLC in IgG4 RD is certainly related with the polyclonal B lymphocytes activation state as proposed in autoimmune disease [8]. When comparing with mean values previously reported in these diseases, the values observed in IgG4-RD patients, measured here, were higher. Although the comparison is not statistically allowed, patients evaluated with the same technique for sFLC measurements in primary Sjögrens syndrome (mean ± SE: *κ* sFLC 16.3 ± 1.4 mg/L and *λ* sFLC 19.3 ± 1.5 mg/L) had much lower values than those reported here [8]. These values were also higher than those observed in rheumatoid arthritis [8] and SLE [7]. This is also observed with the values obtained by the sum of *κ* + *λ* FLC with a median of 71.28 mg/L (36.33–126.9) in our cohort as compared with studies in SLE (median 48.3 mg/L (30.0–70.1)) and RA (median 32.3 mg/L (22.0–44.9)) [7]. These findings could suggest that the increase of sFLC in IgG4-RD is more marked than in other diseases associated with polyclonal B activation. This should be confirmed by future studies. 

An interesting result of our study is that about half (43%) of the IgG4-RD patients have an increased and abnormal *κ* : *λ* ratio. This was not observed when studied in primary Sjögren syndrome, SLEs or RA, despite the increase of sFLC [7, 8]. Usually an abnormal ratio is associated to a context of a monoclonal gammopathy [4]. Abnormal ratios here reflected a proportionally more important increase of the *κ* than the *λ* sFLC. This is probably related to an over production of *κ* FLC in IgG4-RD rather than a decrease of the *κ* FLC clearance because the increase is also significant in patient without renal insufficiency. One explanation could be related to the fact that there is a preferential association of the *γ*4 heavy chain with the *κ* light chain as previously reported in large series of monoclonal IgG4 gammopahties [17, 18]. This is also suggested by the fact that we found a positive correlation of total IgG levels with *κ* sFLC (Spearman *r* = 0.67, *P* = 0.006) but not with *λ* sFLC (Spearman *r* = 0.27, *P* = 0.3). This should be further investigated by analyzing the *κ* and *λ* ratio on IgG4-positive plasmocytes in tissues of IgG4-RD patients. 

However, the absence of a significative correlation between IgG4 and sFLC levels does not confirm this hypothesis. One limit for the interpretation of this last result is the absence of a systematic analysis for the prozone effect for IgG4 measurements in our study [19]. A prozone effect could explain “false low” IgG4 levels in IgG4-RD patients, and thus explain the absence of correlation with sFLC levels. Another explanation for the absence of correlation with IgG4 levels but with total IgG levels is that not only IgG4 are elevated in IgG4 RD but also other IgG subclasses [14] and this could account for the increase of *κ* and *λ* FLC levels. 

No statistical difference depending on the state of IgG4-RD was found in this study but the few samples available for this comparison do not allow any conclusion. A relationship between sFLC concentrations and disease activity measured by the Disease Activity Score 28 has been shown in RA patients [8, 11, 20]. Correlation with disease activity has also been studied in SLE patients whose sFLC levels (in addition to complement C3) were strongly correlated with the SLE disease activity index (SLEDAI) and modified SLEDAI [7]. Larger study involving IgG4-RD patients should assess if the *κ* and *λ* sFLC levels correlate with disease activity as it has been shown in some autoimmune diseases. The comparison of FLC values and *κ* : *λ* ratio in three patients between active and inactive disease did not allow any conclusion here and should be assessed prospectively on a larger study.

Both together the increase of sFLC level, especially the *κ* sFLC, and the *κ* : *λ* ratio could correlate with the volume of the polyclonal “tumoral” infiltrate of IgG4-RD. FLC levels in patients with 3 or less organ involved were lower than in patients with more than 3 organ involved, however this was found not significant. 

We noted in our study that two patients presented with very high sFLC levels and an abnormal *κ* : *λ* ratio. These two patients had *κ* sFLC over 1000 mg/L and a *κ* : *λ* ratio over 5. Both patients also had increased *λ* sFLC over 300 mg/L. Patients presented both with renal insufficiency one of these was requiring hemodialysis and the other one had a glomerular filtration below 30 mL/min. Thus renal insufficiency probably accounted in a large part for FLC increase in these patients. Two other patients of the cohort also presented renal insufficiency but much lower sFLC levels. An increase of sFLC levels is reported in renal insufficiency [1, 21] and is related to a decrease of FLC clearance. However in this condition the *κ* : *λ* ratio is only slightly increased except in the case of a monoclonal gammopathy [21]. Interestingly one of these two patients (no. 7) was diagnosed three month after the sFLC biological evaluation showing an abnormal *κ* : *λ* ratio with a high grade B lymphoma. He was known for a 10 year history of a histologically proven IgG4 related pancreatitis and was reevaluated at the time of sFLC evaluation for a relapse of IgG4-RD after corticosteroid discontinuation with kidney and lymph node involvements (both histologically proven with accurate pathological criterias of IgG4-RD [14]). Lymphoma has been previously reported in some patients with IgG4-RD [22–24]. This single observation suggest that IgG4-RD patient with very high levels of sFLC and an abnormal *κ* : *λ* ratio should be carefully screened and followed for the risk of developing lymphoma. 

## 5. Conclusion

In this study, we show that patients with IgG4-RD with or without renal insufficiency have increased sFLC levels. This is probably related to an increase of FLC production as previously reported in some autoimmune diseases. The sFLC increase is mainly related to an increase of the *κ* FLC. This is associated in 43% of patients with an abnormal increased *κ* : *λ* ratio that is usually not observed in other situations of polyclonal B lymphocyte activation even when associated with impaired renal function. We observed in one case that a major increase of these values precedes the occurrence of a lymphoma complicating an IgG4-RD. In this retrospective study of 16 patients, we could not assess the correlation of sFLC values or *κ* : *λ* ratio with disease activity and patient's disease characteristics, that will require larger and prospective studies. These results pave the way to analyze the values of sFLC in IgG4-RD and their utility in patients followup. 

## Figures and Tables

**Table 1:** Patients' characteristics.

*N*	Age, gender	IgG4-RD organ involvement	*κ* FLC mg/L	*λ* FLC mg/L	*κ*/*λ* ratio	GFR	Disease status
1*	84, M	RPF, mesenteric IPT, lung, LN	59.85	29.63	2.02	—	A
2	71, M	Kidney, LN, pancreas, biliary tree, prostate	41.97	29.31	1.43	39	A
3	63, M	RPF, AIP, sclerosing cholangitis, sialadenitis, renal IPT, LN, periaortitis, pleura/lung	49.8	**24.54**	2.02	—	A
4	71, M	RPF, aortitis, dacryoadenitis, sialadenitis, LN	27.55	47.2	0.58	—	A
5*	59, M	RPF, aortitis, carotidal and iliac arteritis, LN, hypophysitis	30.48	**9.48**	3.21	—	A
6*	74, M	AIP, orbital IPT, sialadenitis, sclerosing cholangitis, sclerosing cholecystitis, TIN	**12.44**	**12.3**	1.01	—	A
7	75, M	AIP, LN, TIN, sialadenitis, pleura/lung	2610	511	5.11	12	A
8	63, M	Meningeal IPT	20.9	**18.4**	1.14	—	A
9	67, M	Periaortitis	20.39	**12.46**	1.63	—	A
10	65, M	AIP	61.85	65	0.95	—	A
11	58, F	Pulmonary IPT	20.14	**16.19**	1.24	—	A
12	27, M	AIP, sclerosing cholangitis, LN	219.62	33.24	6.6	—	A
13	80, M	Sialadenitis, TIN, LN, lung	1501	312	4.84	6	A
14	62, M	Parotid IPT, LN	**13.8**	**13**	1.06	—	A
15	76, M	RPF, LN	46.2	**22.1**	2.09	—	A
16	84, M	TIN, LN, PAI, RPF, sialadenitis	97.3	79.42	1.22	67	NA
		Mean**	*315.7 *	*77.06 *			
		Median**	*41.97 *	*24.54 *	*1.63 *		
		IQR**	*(20.39–61.85)*	*(13–47.2)*	*(1.06–3.2)*		

Values in bold are normal values.

*Patients from which samples were analyzed two times (in active and nonactive disease). Given values in the table are values during active disease, **given values for mean, median and IQR are calculated with values obtained in patients with active disease (patient no. 16 is excluded). AIP: autoimmune pancreatitis; IPT: inflammatory pseudotumor: LN: lymph nodes; RPF: retroperitoneal fibrosis; TIN: tubulointerstitial nephritis; GFR: glomerular filtration rate (mL/min/1.73 m^2^). Disease status: A: active, NA: nonactive.
